# Measurement of the Fetal Ear Length Has No Clinical Value

**DOI:** 10.3390/jcm12093084

**Published:** 2023-04-24

**Authors:** Sławomir Witkowski, Maria Respondek-Liberska, Rafał Zieliński, Iwona Strzelecka

**Affiliations:** 1 Department of Prenatal Cardiology, Polish Mother’s Memorial Hospital Research Institute, 93-338 Lodz, Poland; slawek86@gmail.com (S.W.);; 2 Medical Faculty, Ludwik Rydygier Collegium Medicum Bydgoszcz, 85-067 Bydgoszcz, Poland; 3 Department of Diagnoses and Prevention of Fetal Malformations, Medical University of Lodz, 90-419 Lodz, Poland; 4 Collegium Medicum, Jan Kochanowski University Kielce, 25-369 Kielce, Poland; 5 Department of Otorhinolaryngology, Provincial Polyclinic Hospital in Kielce, 25-736 Kielce, Poland

**Keywords:** fetal ear, extracardiac anomalies, ultrasound, prenatal cardiology, fetal anomalies

## Abstract

The long-term monitoring of a fetus with genetic and non-genetic anomalies is still a challenge for prenatal medicine. Ultrasound screening must be based on some measurement ranges, which show the trend of development of fetal body parts in a given period of time. One of them is the fetal ear auricle. This study presents an analysis of the fetal ear auricle length in healthy fetuses to establish normal ranges of fetal ear auricle length. The study group included 132 healthy fetuses. The gestational age of healthy fetuses ranged from 17.0 to 39.5 weeks of gestation according to LMP. The range of fetal ear length measurement was 10.00–40.00 mm with an average value of 23.49 mm. In the group of fetuses in the second trimester of pregnancy, the range of fetal ear length measurement was 18.00–28.00 mm, whereas in the group of fetuses in the third trimester of pregnancy, the range was 16.00–40.00 mm. In order to check the usefulness of this parameter, an analysis of this marker in fetuses with extracardiac anomalies, including genetic and non-genetic disorders is shown. The fetal ear measurement can fall within the normal range even if there are some genetic or non-genetic disorders. Therefore, the fetal ear measurement does not provide any diagnostic value in terms of detecting any fetal genetic and non-genetic disorders, which is supported by the analysis of the data provided in this study. Our study has proved that measurement of the fetal ear auricle is possible; however, its clinical usefulness for perinatal management is currently very limited.

## 1. Introduction

Extracardiac abnormalities are common in congenital heart diseases as well as in genetic and non-genetic syndromes. Therefore, screening of fetuses is important for the early detection of any syndromes and the appropriate management of these patients. There are many ultrasound markers used for the detection of genetic and non-genetic syndromes [1]. One of them may be the fetal ear auricle. Fetal ear auricle length measurements have already been discussed in the literature in terms of the prediction of aneuploidy, mainly trisomy 21, trisomy 18, triploidy, sex chromosomal abnormalities, and chromosomal abnormalities. These studies showed a shorter ear in fetuses with genetic disorders [2,3]. In this study, the authors aimed to analyze fetal ear auricle length in healthy fetuses to establish normal ranges of fetal ear length in healthy fetuses and use the elaborated normal ranges in reference to a group of fetuses with genetic and non-genetic abnormalities to check if measurement of the fetal ear auricle can constitute a relevant diagnostic marker in this group of fetuses. In order to check the usefulness of this parameter, this study includes an analysis of the measurement of the fetal ear auricle in fetuses with extracardiac anomalies, including genetic and non-genetic disorders.

## 2. Materials and Methods

This was a single-center analysis of a group of fetuses who had fetal ultrasound examinations at the tertiary center from 2019 to 2022. The information on the fetal ear auricle length was collected from the database of our unit (Fetal Pathology of the Medical University of Lodz). All the healthy fetuses were labeled with normal biometry, normal heart anatomy, and normal cardiac function without any extracardiac malformations or extracardiac anomalies. Gestational age (GA) was calculated based on the last menstrual period (LMP). The gestational age of healthy fetuses (n = 132) ranged from 17.0 to 39.5 weeks of gestation according to LMP. The measurements performed did not affect clinical management. We only focused on the analysis and interpretation of the collected data. As a result, approval from the Ethical Committee was not needed. All the patients gave their consent for scientific analyses of their data at our center. The measurement of the fetal ear auricle length was performed by mainly one person at our tertiary center, which made the analysis uniform and correct.

### 2.1. Exclusions

All the patients with any abnormalities other than extracardiac anomalies, i.e., cardiac anomalies (for instance, isolated polyhydramnion, isolated oligohydramnion, 2-vessel umbilical cord, placentitis, and umbilical cord around the fetal neck) were excluded from the first part of the study. 

### 2.2. Fetal Ultrasound Examinations

The ultrasound examinations were performed with the use of the GE Voluson E8, GE Voluson E10, and Philips iU22 ultrasound machines with convex transabdominal transducers. Fetal ear auricle length was measured from the tip of the helix to the end of the lobe in the longitudinal view. This measurement was performed during a detailed fetal ultrasound evaluation and the value was added to 80% of the unit’s database by one physician (MRL).

### 2.3. Statistical Analysis

Statistical analysis was performed using Statistica 13.3 and Excel 365 software. Scatter graphs of fetal ear length measurement in healthy fetuses with categorization into the second and third trimester was created. A box plot with minimal, maximal, and median values was created for comparison of the second and third trimesters of pregnancy in healthy fetuses. An additional graph of the median and interquartile range of the fetal ear length in 5 subgroups according to LMP was prepared (*p* < 0.05). The data were normally distributed. The Kruskal–Wallis test was used to analyze the fetal ear length in subgroups according to LMP. A *p*-value < 0.05 was considered statistically significant. 

## 3. Results

242 fetuses were selected for this study. For the purpose of establishing normal ranges of fetal ear auricle length in healthy fetuses, 110 fetuses were excluded due to the presence of extracardiac anomalies. The study group included 132 healthy fetuses. The gestational age of healthy fetuses (n = 132) ranged from 17.0 to 39.5 weeks of gestation according to LMP.

**Figure 1:**
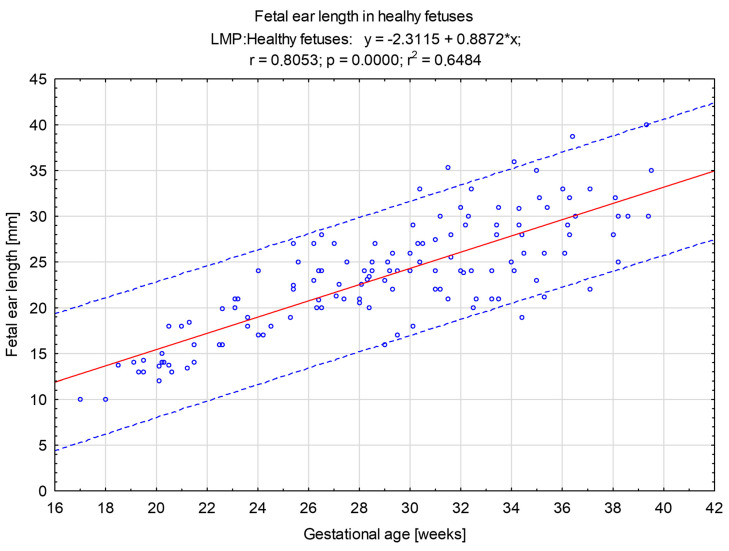
A graph of fetal ear length measurement (in mm) in healthy fetuses (n = 132) in relation to the gestational age (in weeks) with trend lines and percentiles. The red line is 50th percentile, whereas the upper blue line is 90th percentile and the lower blue line is 10th percentile.

The study group included fetuses with normal biometry, normal heart anatomy, and normal cardiac function without any extracardiac malformations or extracardiac anomalies. This group was used for the preparation of a graph of normal ranges of fetal ear length (Figure 1).

**Figure 2:**
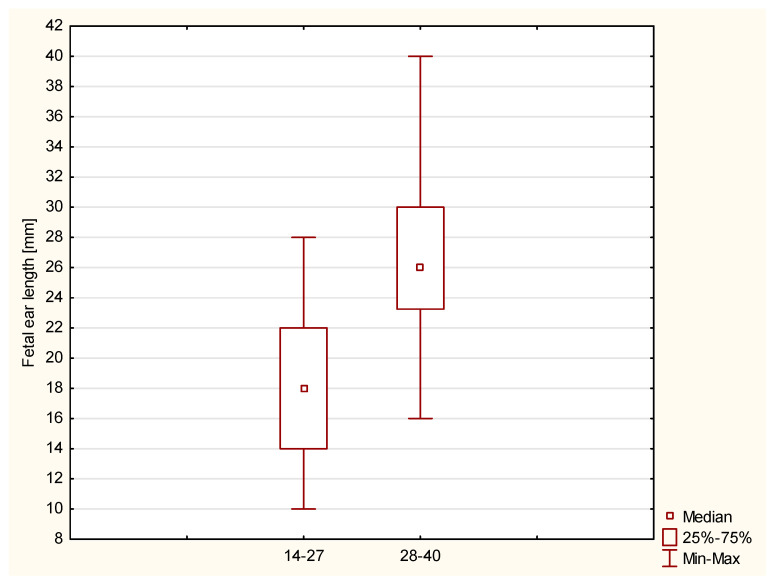
Median and range (with minimum and maximum values) of the fetal ear length in healthy fetuses in the second (14–27 weeks) and third (28–40 weeks) trimesters of pregnancy.

**Figure 3:**
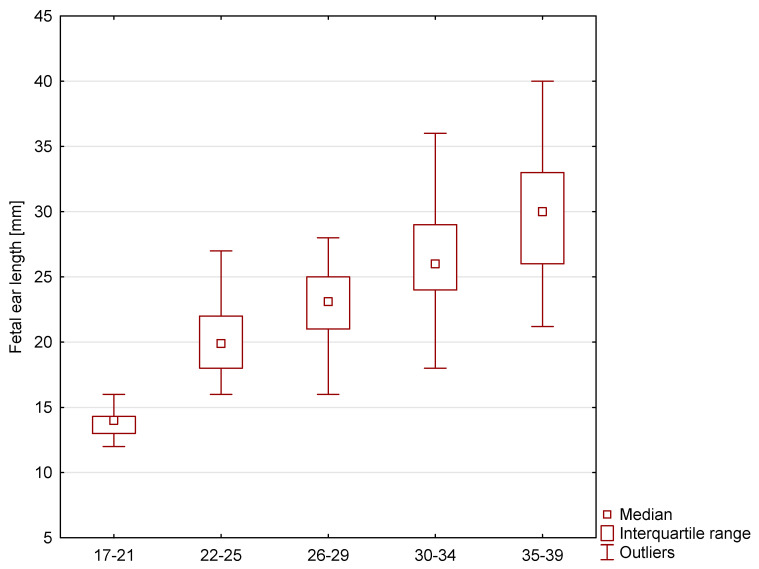
Median and interquartile ranges of the fetal ear length in healthy fetuses in subgroups according to LMP.

In the study group, the range of fetal ear length measurement was 10.00 mm–40.00 mm with an average value of 23.49 mm. The length of 14.00 mm constituted the 10th percentile, and the length of 31.00 mm was the 90th percentile. This group of fetuses was divided into the second and third trimesters of pregnancy. In the group of fetuses in the second trimester of pregnancy, the range of fetal ear length measurement was 18.00 mm–28.00 mm, whereas in the group of fetuses in the third trimester of pregnancy, the range was 16.00 mm–40.00 mm (Figure 2). The R-value of 80.53% and R^2^ value of 64.84% show a good correlation between gestational age and fetal ear length. The variability of the fetal ear length is determined by the gestational age to a good extent. The median and interquartile ranges of the fetal ear length in healthy fetuses in subgroups according to LMP are presented in Figure 3.

**Figure 4:**
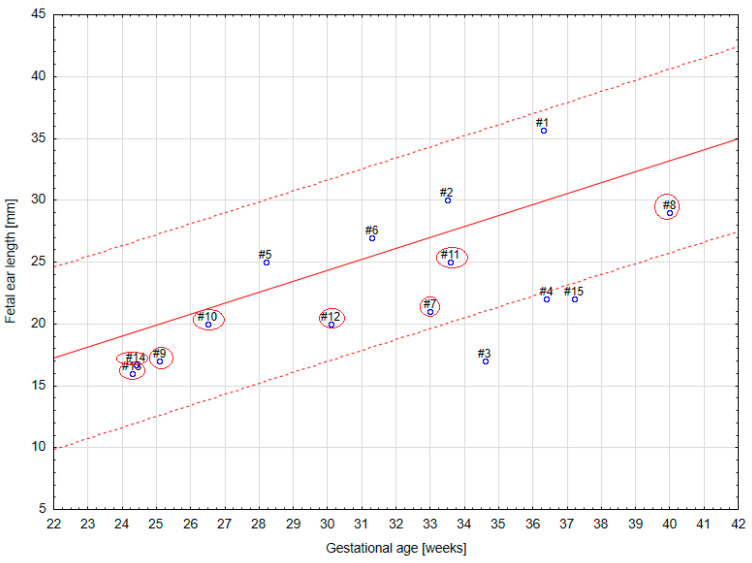
A scatter graph of 15 cases of fetuses from our tertiary center with ear length around the 90th percentile, above and below the 50th percentile, and around and below the 10th percentile on the background of the graph of normal ranges of fetal ear length. Marked with circles are cases of fetuses with Down syndrome.

Based on the elaborated normal ranges of fetal ear length in healthy fetuses, some fetuses beyond the normal range of fetal ear length (below and above the 50th percentile, around 90th percentile, and around 10th percentile) were randomly selected for further analysis. These fetuses presented extracardiac anomalies and genetic or non-genetic disorders. In one fetus the parents presented ear dysplasia. These fetuses were shown on the background of a scatter graph (Figure 4) with normal ranges of fetal ear length. A summary of randomly selected fetuses is presented in Table 1 below. Ultrasound examinations of the fetal ear auricle are presented in Figures 6–10, including 2D and 3D examinations and an image of a newborn with ear defects.

Table 1 shows that fetal ear length can be affected by many different extracardiac anomalies as well as genetic and non-genetic disorders. Any discrepancies in the ultrasound measurement of fetal ear length (too large or too small) according to the literature should be a warning sign for an ultrasound specialist of the possibility of occurrence of some other disorders in the fetus and should require further monitoring and diagnosis.

**Figure 5:**
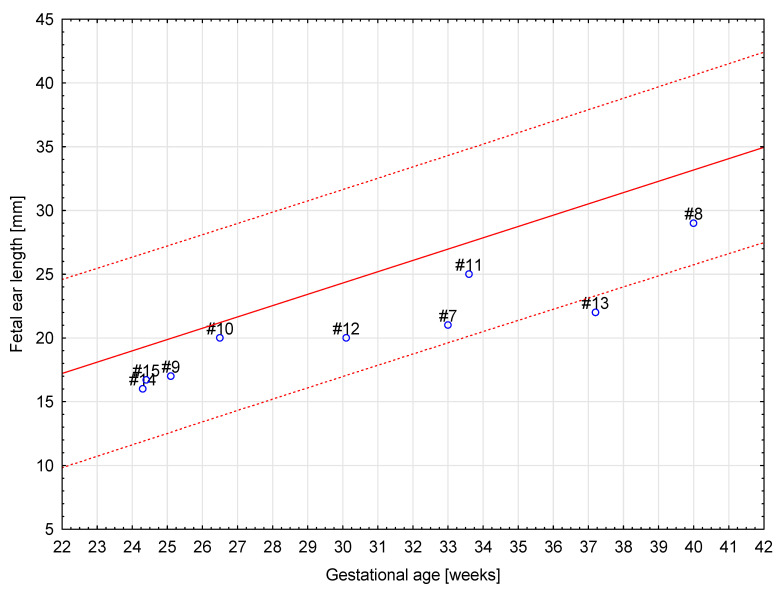
A scatter graph of eight cases of fetuses (#7–#14) from our tertiary center with Down syndrome and one case of a fetus with Edwards syndrome (#15).

Figure 5 confirms that fetuses with genetic disorders, such as Down syndrome and Edwards syndrome, may have ear lengths below the 50th percentile.

## 4. Discussion

**Figure 6:**
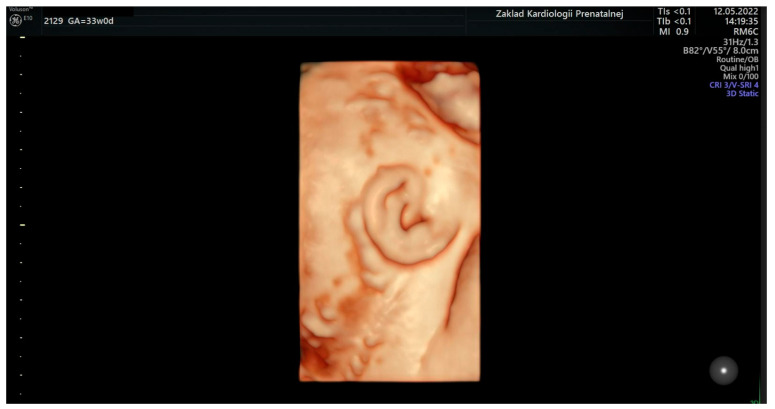
An image of a fetal ear in 3D ultrasound examination. Measurement of the fetal ear at the gestational age of 33 weeks.

**Figure 7:**
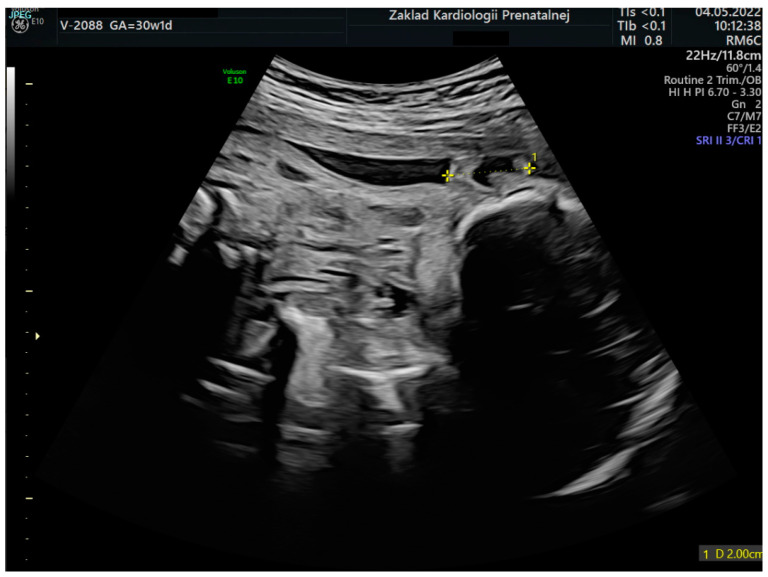
An image of a fetal ear in 2D ultrasound examination. Measurement of the fetal ear at the gestational age of 30 weeks and 1 day.

**Figure 8:**
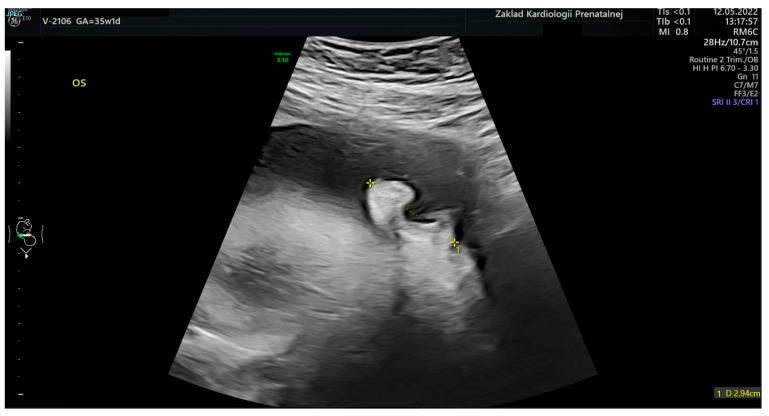
An image of a fetal ear in 2D ultrasound examination. Measurement of the fetal ear at the gestational age of 35 weeks and 1 day.

**Figure 9:**
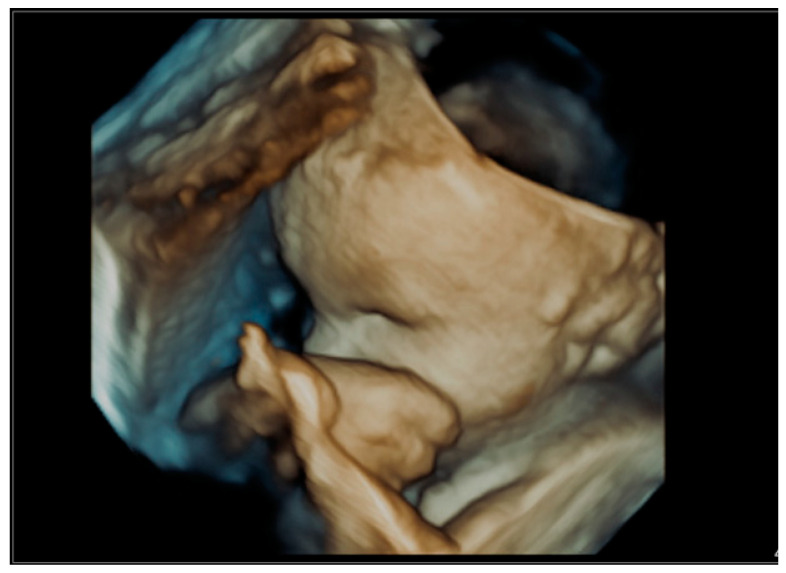
An image of a fetal ear auricle in 3D ultrasound examination at the gestational age of 35 weeks and 1 day. A genetic test of the amniotic fluid: the use of whole genome oligonucleotide microarrays (CytoSure Constitutional v3 (8x60k), Oxford Gene Technology, GRCh37/hg19) with an average resolution of 120 kpz–arr(X,Y)x1,(1-22)x2–No abnormalities were detected.

**Figure 10:**
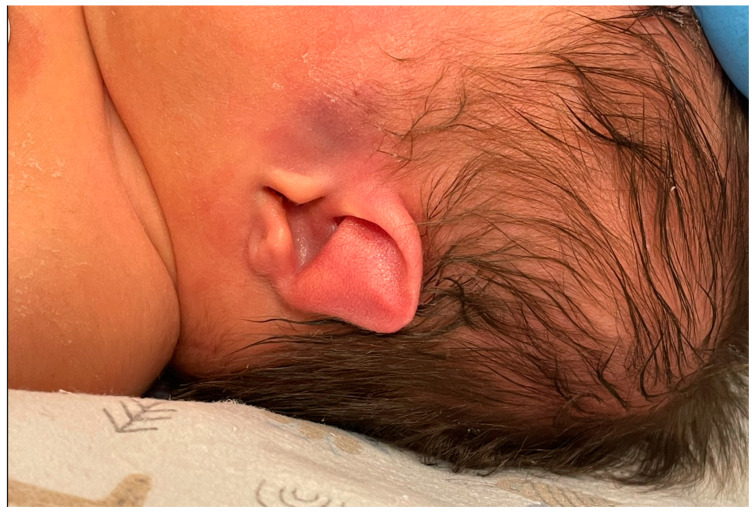
A postnatal image of an ear auricle from Figure 9. The 2D measurement was within normal range; however, the shape of the ear auricle is grossly abnormal.

The ear is an anatomical structure which consists of three distinct structures: the inner ear, the middle ear, and the outer ear. During embryogenesis, the ear starts to develop from around the 22nd day of prenatal life and is fully developed by the 18th week of gestation [4]. The fetal external ear shape is similar to an adult, but its prenatal structure may be quite complex, and thus, sometimes difficult to be observed during prenatal ultrasound examination [5]. Embryogenesis is the stage where the external ear is developed. The ear is visualized most often as a hyperechogenic structure in ultrasound examinations and is most often observed in the longitudinal plane [6]. The structure of the fetal ear which is observed during ultrasound examination is the ear auricle in the longitudinal plane. A sample visualization of the fetal ear during a 3D ultrasound examination can be found in Figure 6, Figure 7, Figure 8, Figure 9 and Figure 10. Visualization of the fetal ear is not quite easy, and it is not recommended in basic screening in obstetrical ultrasound. However, it is possible to observe such an organ in ultrasound examination; therefore, ultrasound specialists may analyze the structure of the fetal ear and measure the fetal ear length. Observation of both ears may be used to compare their structure and shape. In our work, we focused on the analysis and measurement of only one ear after making sure that both ears are present. The choice of left or right ear depended on the position of the fetus and was simply random. The fetal ear can be observed in ultrasound in the second and third trimesters, but the best period of its analysis in terms of any abnormalities and malformations is the second trimester of pregnancy [7,8,9,10]. The development of the ear may be affected by karyotype anomalies and genetic syndromes [11]. The fetal ear length measured during ultrasound examination has already been the subject of some medical articles which focus on the creation of normal ranges of fetal ear length. When analyzing the normal ranges presented in the literature [6,12,13], it can be shown that the ranges are consistent with the normal ranges of the fetal ear length shown in this paper based on the data from our tertiary center. The available literature focuses mainly on the assessment of the fetal ear in terms of aneuploidy, such as trisomy 13, 18, and 21, and finds quite a statistically significant correlation between aneuploidy and smaller ears in fetuses observed in prenatal life [14,15,16,17,18]. According to them, small ears can constitute a useful tool in the prediction of aneuploidy [19]. Abnormally enlarged ears can be a marker of the trisomy 4p syndrome in which a fetus presents ‘a characteristic facial appearance, postnatal growth retardation, severe psychomotor retardation with or without seizures, and microcephaly [20]. Different craniofacial malformations such as auriculocondylar syndrome, which is characteristic of micrognathia, mandibular condyle hypoplasia, and auricular abnormalities, may affect the length of the fetal ear auricle [21]. The other syndromes and anomalies where the fetal face, including the ear, is affected include the Fryns syndrome [22] or the CHARGE syndrome [23,24]. There can be a relationship between the evaluation of the inner ear of the fetus and congenital cytomegalovirus infection [25]. Our study attempted to compare the findings with the data from our center and we managed to conclude that even if the above-mentioned malformations and anomalies can affect the length of the ear, our analysis and presented ranges show that the measurement of the fetal ear in this group of fetuses may fall within our elaborated normal ranges.

There are plenty of possibilities for taking measurements additional to routine fetal biometry, such as fetal stomach and urinary bladder [26].

The aim of our study was to evaluate the usefulness of prenatal measurements of fetal ear length in a tertiary center. The study proves that even though visualization and measurement of the fetal ear are possible, it is very rarely used on a daily basis. The results of this paper suggest that measurement of the fetal ear can be applied in daily practice, but it has a limited practical value as shown in Figure 4.

The range of fetal ear length measurement in healthy fetuses in the gestational age of 17.0 to 39.5 weeks according to the last menstrual period is 10.00–40.00 mm. In the second trimester of pregnancy, the range of fetal ear length measurement is 18.00–28.00 mm, whereas in the third trimester of pregnancy, the range is 16.00–40.00 mm. The R-value of 80.53% and R^2^ value of 64.84% show a good correlation between gestational age and fetal ear length. What must be emphasized is that the fetal ear measurement can fall within the normal range even if there are some genetic or non-genetic disorders. That is why the fetal ear measurement does not provide any diagnostic value in terms of detection of any fetal genetic and non-genetic disorders. A good example is presented in Figure 9 (prenatal imaging) and Figure 10 (postnatal follow-up). This is an example of a newborn with an abnormal shape of the fetal ear auricle even though the newborn does not present any genetic abnormalities, which was confirmed in a genetic test of the amniotic fluid at the 22nd week of pregnancy with the use of the whole genome oligonucleotide microarrays (CytoSure Constitutional v3 (8x60k), Oxford Gene Technology, GRCh37/hg19) with an average resolution of 120 kpz–arr(X,Y)x1,(1-22)x2–correct result (test number: aCGH-PD10038, DNA number: PD10038). The genetic test was performed at a leading genetic clinic in Poland. This confirms our conclusion that fetal ear measurement has no clinical value. Even if fetuses with Down syndrome have a smaller fetal ear [2,3] compared to fetuses without any disorders, in this study, the authors do not support such observations in relation to fetuses with trisomy 21.

It is also important to take into consideration the possibility of visualizing both ears during prenatal ultrasound examination, especially when a parent presents agenesis of one of the ears or both ears. However, the visualization of the ears depends on the fetal head position, and in some cases, it may not be possible to obtain a proper image of the fetal ear or ears. The fetus can be moving all the time during the ultrasound examination, and it can be difficult for some practitioners to correctly measure the fetal ear auricle.

There are some important clinical implications that arise from our study. This can be a time-consuming procedure and, therefore, may not be used on a regular basis in regular examinations. However, in tertiary centers, where a scrutinized diagnosis is needed, it is important to measure all the parts of the fetal body regardless of the time consumed.

A key strength of this paper is that it uses quite a large national dataset of healthy fetuses, based on which a graph of normal ranges was elaborated and a study group was formed, which was thoroughly selected based on complete data about the fetuses and newborns. This study shows that even if a genetic test is performed and its results are correct, the ultrasound measurements and visualizations can demonstrate that some anomalies may still be present. This is in contrast to previous data analyses conducted on a large group of fetuses. As a result, the authors conclude that even if genetic or non-genetic anomalies are present, the measurements of the fetal ear auricle can fall within the normal range.

In spite of having a large database of measurements of fetuses, there are also some limitations that need to be considered. The most important one is a small group of fetuses in the study group. However, this group can be extended for further studies. Another limitation is the fact that ultrasound examination of the fetal ear auricle can be difficult and time-consuming.

## 5. Conclusions

Our study has proved that measurement of the fetal ear auricle is possible; however, its clinical usefulness for perinatal management is currently very limited.

## Figures and Tables

**Table 1:** A summary table of randomly selected samples of 15 cases of fetuses with ear length.

Case No.	Fetal Age According to LMP [Weeks]	Fetal Ear Length [mm]	Extracardiac Anomalies and Genetic or Non-Genetic Disorders	Interpretation of Fetal Ear
1	36.3	35.7	Mother with hearing deficiency, father with ear agenesis	Around 90th percentile
2	33.5	30.0	Multicystic dysplastic kidney	Above 50th percentile
3	34.6	17.0	Microcephaly	Around 10th percentile
4	36.4	22.0	Hydrocephalus	Below 50th percentile
5	28.2	25.0	Craniorachischisis, cleft palate with cleft lip, esophageal atresia	Above 50th percentile
6	31.3	27.0	Dandy–Walker syndrome	Above 50th percentile
7	33.0	21.0	Down syndrome	Around 10th percentile
8	40.0	29.0	Down syndrome	Below 50th percentile
9	25.1	17.0	Down syndrome	Below 50th percentile
10	26.5	20.0	Down syndrome	Around 50th percentile
11	33.6	25.0	Down syndrome	Below 50th percentile
12	30.1	20.0	Down syndrome	Around 10th percentile
13	24.3	16.0	Down syndrome	Below 50th percentile
14	24.4	16.7	Down syndrome	Below 50th percentile
15	37.2	22.0	Edwards’ syndrome	Below 10th percentile

## Data Availability

Not applicable.
